# Effects of Acupuncture on Hospitalized Patients with Urinary Retention

**DOI:** 10.1155/2020/2520483

**Published:** 2020-01-19

**Authors:** Suhui Chen, Hua Sun, Hong Xu, Yamin Zhang, Huanyuan Wang

**Affiliations:** Department of Traditional Chinese Medicine, Peking Union Medical College Hospital, Chinese Academy of Medical Sciences, Beijing 100730, China

## Abstract

**Objective:**

The aim of this study was to investigate the effects of acupuncture on urinary retention and provide treatment suggestions.

**Methods:**

A total of 113 hospitalized patients with urinary retention were included in this study. The GV20, CV6, CV4, CV3, ST28, SP6, and SP9 points were selected as the main acupoints. Acupuncture therapy was conducted for 30 minutes per session. The total number of treatment sessions was determined by the symptoms and the length of hospital stay. Bladder postvoid residual urine volume (PVR) was measured pretreatment and posttreatment by ultrasonic. Efficacy defined as spontaneous urination and a residual urine volume <50 mL was measured.

**Results:**

The median number of acupuncture treatment sessions was 3 (range, 1–12 times). Acupuncture treatment significantly reduced the PVR (545.1 ± 23.9 mL vs 67.4 ± 10.7 mL; *p* < 0.001). Among the 113 patients, 99 (87.6%) patients were cured and 8 (7.1%) patients were improved of their urinary retention. The remaining 6 (5.3%) patients' urinary retention did not improve. The effective rate was 94.7%. There was significant difference in the efficacy rate between patients with one urinary catheterization and with two or more. Acupuncture treatment was not associated with side effects.

**Conclusion:**

Acupuncture is an effective and safe treatment option for urinary retention. Early application of acupuncture treatment should be considered in clinic, and repeated urinary catheter insertion and removal should be avoided. Our study suggests that a randomized controlled study with a large sample size to verify the efficacy of acupuncture for the treatment of urinary retention is warranted.

## 1. Introduction

Urinary retention refers to impaired voiding despite a full bladder, which leads to an elevated postvoid residual urine volume (PVR) [1]. It is a frequent result of surgery or diseases that can lead to urinary tract infection and increase hospital stays. However, there is no consensus definition for urinary retention, either postoperatively or otherwise. The incidence of urinary retention is reported to be from 3 to 43% [2–5].

Although urinary retention is not a life-threatening condition, it causes great pain to the patient. Urinary retention may lead to excessive bladder expansion and permanent detrusor injury. If not treated in time, urinary retention may lead to secondary urinary tract infection, reflux nephropathy, bladder rupture, kidney failure, and other serious consequences [6–8]. Currently, indwelling catheters are often used to treat urinary retention in clinical practice; however, patients may find catheterization to be distressing, undignified, and uncomfortable. Furthermore, prolonged indwelling catheters increase the chance of urinary tract infection and the length of hospital stay [9, 10].

There are few large-sample studies on the treatment of urinary retention by acupuncture. Previous studies mainly focused on a specific disease, such as urinary retention after gynecological operation or postpartum. Whether there are differences in the efficacy rate of acupuncture on urinary retention caused by different causes and whether there are differences in patients with one urinary catheterization and with two or more have not been well studied.

This study retrospectively analyzed 113 urinary retention hospitalized patients who were treated with acupuncture consultation in general hospital during the past five years. The results suggest that acupuncture is an effective treatment for urinary retention. Furthermore, the data suggest that a prospective randomized clinical trial with large sample size is warranted.

## 2. Methods

### 2.1. Study Design

This study was a retrospective record review of hospitalized patients with urinary retention who received acupuncture consultations in the Department of Traditional Chinese Medicine in Peking Union Medical College Hospital (PUMCH) from July 2013 to November 2018. Patient data, including age, sex, disease causes, duration of urinary catheterization, frequency of urinary catheter reinsertion, PVR, and urinary tract infection, were obtained from Hospital Information System (HIS).

The study only involved the collection of existing data from HIS database of PUMCH. Because these data were deidentified, retrospective, the Institutional Review Board (IRB) of PUMCH has determined that no further review was required (protocol number: S-K 774).

### 2.2. Participants

Patients who had normal blood coagulation function and were willing to undergo acupuncture treatment received the treatment. The diagnosis of urinary retention was established by the attending physician and defined as an inability to urinate spontaneously or difficulty in complete micturition with a bladder postvoid residual urine volume (PVR) >200 mL requiring a bladder urinary catheter or a need for urethral catheterization as indicated by an ultrasound examination [11, 12].

### 2.3. Study Treatment

**Figure 1:**
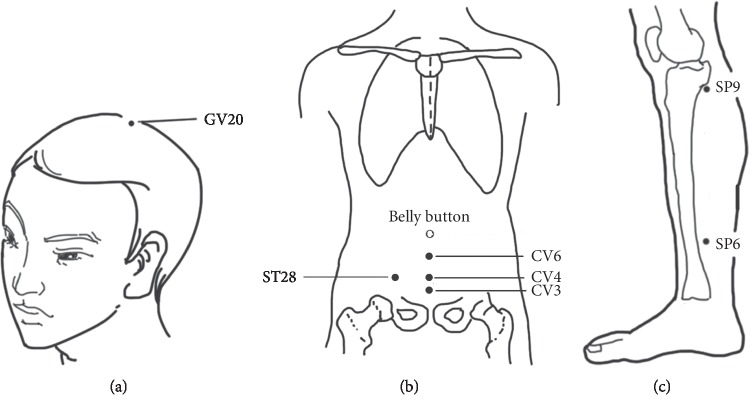
The nomenclature and location of the main acupoints. (a) GV20. (b) CV6, CV4, CV3, and ST28. (c) SP6 and SP9.

Acupuncture treatments were performed by three experienced acupuncturists licensed in traditional Chinese medicine (TCM) who were responsible for the acupuncture consultation during the study period. All three acupuncturists had eight years of training in acupuncture and TCM and more than 3 years of clinical experience. Acupuncture point selection was based on TCM meridian theory and clinical experience. The main acupoints included the GV20, CV6, CV4, CV3, ST28, SP6, SP9, and auricular (bladder and urethra) points. Supplementary acupoints were established as follows: ST36 for qi deficiency, LR3 for liver qi stagnation, and KI3 for kidney essence deficiency. The acupoints applied in this study were located according to *the Nomenclature and Location of Acupuncture Point* of National Standard of People's Republic of China (GB/T 12346-2006).  Figure 1 shows the nomenclature and location of the main acupoints.

Patients rested in the supine position and relaxed the body, and the catheter was opened to empty the urine in the catheter before the acupuncture treatment. If the catheter had been removed, acupuncture began immediately. The acupuncturist routinely disinfected the skin of the acupoints and used disposable sterile needles (0.30 mm × 40 mm). The acupuncture points were punctured 10–30 mm deep depending on the point selected. The needles were maintained for 30 min after achieving deqi and were manipulated once every 10 min. Deqi is also called needle sense, which is the induction of meridian qi produced by inserting acupuncture into acupoints. When qi comes, both the patient and the acupuncturist can feel. For the patient, deqi is the sensation of acid, numbness, distension, heaviness under the acupoints, or the sensation of diffusion and conduction in a certain direction, and for the acupuncturist, deqi is the sensation of sinking and tightening under the needle, moderate manipulation was applied until the patient and the acupuncturist had the sensation of deqi. Point GV20 was punctured quickly at a 15° angle along the scalp skin, and the needle was rotated with a torsional amplitude of approximately 180° at a frequency of approximately 200 times/min. Points CV6, CV4, CV3, and ST28 are on the lower abdominal, which were punctured at a depth of 20–30 mm, and the needle tip pointed toward the perineum. The acupuncturist did not puncture too deeply to avoid damaging the bladder. A gentle manipulation of lifting, thrusting, and rotating was used to promote deqi until patients had a sense of numbness, distension, and radiation in the perineum. Points SP6, SP9, ST 36, LR3, and KI3 were punctured vertically.

Patients who received only one treatment session versus those who received more depended on the degree of injury and the length of hospital stay. If the urinary catheter had been removed, acupuncture began immediately, 30 minutes for one treatment session; patients who can void spontaneously after receiving one treatment session and had qualified PVR did not need to receive acupuncture anymore, or else, they need to insert the urinary catheter and receive more treatment sessions. There are usually 3 treatment sessions as a period of treatment; after 3 treatment sessions, clip the catheter and if patients felt the sensation of a fulling bladder, try to remove the urinary catheters and see if they can urinate successfully, and if the sensation of a fulling bladder was not obvious or patients cannot urinate successfully after the removal of the urinary catheters, they had to receive for more treatment sessions.

### 2.4. Outcome Measures

The outcome measures include clinical symptoms and PVR [13–15]. The PVR was measured by bedside ultrasound examination performed by the ward physician or sonographer. Cured was defined as the ability to urinate spontaneously without symptoms and signs or with a voided volume ≥200 mL and PVR <50 mL or 50 mL < PVR < 100 mL for elderly patients (age ≥65 years old). Improved was defined as (1) improvement of symptoms and (2) a PVR >100 mL or a reduction of PVR by >200 mL. Unchanged was defined as no change in symptoms and PVR.

### 2.5. Statistical Analysis

All data analyses were performed using SPSS 17.0 (SPSS Inc. Chicago, IL) statistical software. A descriptive analysis of the data was performed using the number and percentages for categorical variables and the median (range) or mean ± SEM for continuous variables. The PVR was compared before and after treatment. Paired sample *t*-tests were utilized for continuous variables, and a value of *p* < 0.05 was considered statistically significant.

## 3. Results

### 3.1. Characteristics of Patients with Urinary Retention

A total of 113 hospitalized patients with urinary retention were included in this study. All of them had been treated with abdominal warming, listening to water, or injecting neostigmine. The patients were either unable to urinate by themselves within 6 hours after catheter removing or able to urinate but with many residual urines in the bladder during monitoring. The patient characteristics including age, sex, duration of urinary catheterization, number of urinary catheter reinsertions, and accompanying urinary tract infections, are shown in Table 1. Most of the patients cannot void after a period of rest, and the median duration of urinary catheterization was 12 days (range, 1–50 days). Urinary retention requiring repeated reinsertion of the urinary catheter occurred in 109 patients (96.5%), and the mean frequency of urinary catheter reinsertion was 2 (range, 0–6 times). Urinary catheter reinsertion more than twice occurred in 57 patients (50.4%), and reinsertion more than 3 times occurred in 19 patients (16.8%) before acupuncture treatment. Among the 113 hospitalized patients, 27 (23.9%) experienced urinary tract infection owing to the indwelling catheter.  Table 2 lists the urinary retention causes, including gynecological surgery, obstetric delivery, myelopathy, and lumbar spine surgery. Gynecological surgery and obstetric delivery accounted for 87.6% of the urinary retention cases. Among these two causes, there were 78 cases of gynecological surgery (including 66 cases of gynecological malignant tumor surgery, 9 cases of gynecological benign tumor surgery, and 3 cases of pelvic organ prolapse surgery) and 21 cases of obstetric delivery (including 20 cases of vaginal delivery and 1 case of cesarean delivery). Among the 20 cases of vaginal delivery, 19 (95.0%) cases included left mediolateral episiotomy and 11 (55.0%) cases involved prenatal urinary retention.

### 3.2. Acupuncture Treatment

**Figure 2:**
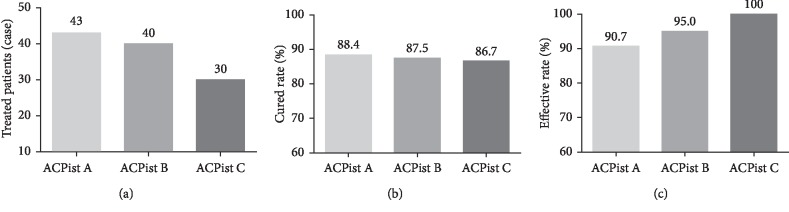
Characteristics of three acupuncturists involved in this study. (a) The treated patients, (b) the cured rate, and (c) the effective rate of three acupuncturists involved in this study. The data shown are the number and percentages (note: ACPist, acupuncturist).

**Figure 3:**
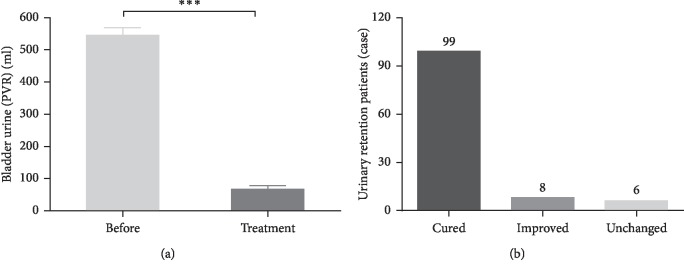
Effects of acupuncture on patients with urinary retention. (a) The changes in bladder postvoid residual urine volume (PVR, ml) after acupuncture treatment. The data shown represent the mean ± SEM, ^*∗∗∗*^*p* < 0.001. (b) The outcome of patients who received acupuncture treatment. The data shown represent the number of cases.

**Figure 4:**
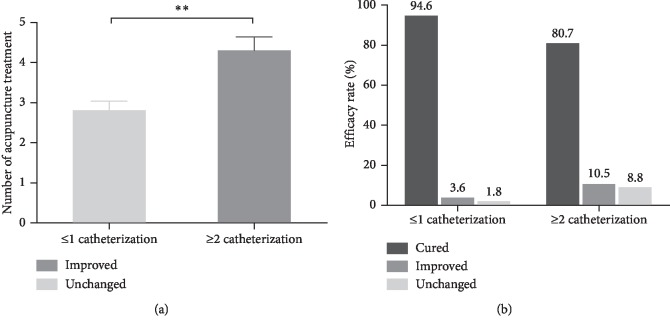
Therapeutic effect between acupunctured patients with one urinary catheterization and with two or more. (a) The number of acupuncture treatment between patients with less than one urinary catheterization and with two or more. The data shown represent the mean ± SEM, ^*∗∗*^*p* < 0.01. (b) Efficacy rate of patients with one urinary catheterization and with two or more. The data shown as percentages.

**Figure 5:**
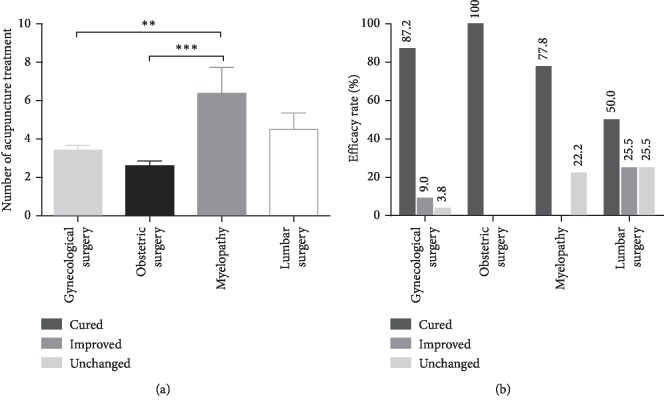
Therapeutic effect among acupunctured patients with gynecological surgery, obstetric surgery, myelopathy, and lumbar surgery. (a) The number of acupuncture treatment among patients with gynecological surgery, obstetric surgery, myelopathy, and lumbar surgery. The data shown represent the mean ± SEM, ^*∗∗*^*p* < 0.01, and ^*∗∗∗*^*p* < 0.001. (b) Efficacy rate of patients with gynecological surgery, obstetric surgery, myelopathy, and lumbar surgery. The data shown as percentages.

Three experienced acupuncturists were involved for these 113 patients during their consultation period. The number of treated patients for each acupuncturist was 43, 40, and 30. The cured rate among them was 88.4%, 87.5%, and 86.7%, and the effective rate was 90.7%, 95%, and 100%. There was no obvious difference in the degrees of success among the acupuncturists (Figure 2). The median number of acupuncture treatment sessions was 3 (range, 1–12 times). As shown in Figure 3, the PVR was 545.1 ± 23.9 mL before treatment and 67.4 ± 10.7 mL after acupuncture treatment. Acupuncture treatment significantly reduced the PVR (*p* < 0.001). Ninety-nine (87.6%) patients pulled out urinary catheter successfully. Among the cured patients, 75 patients received three or less treatment sessions with 10 patients receiving only one treatment session. Eight (7.1%) patients showed some improvement, while 6 (5.3%) patients did not have improvement. The overall effective rate was 94.7%. In addition, there was significant difference in the efficacy rate between patients with one and two or more urinary catheterization (Figure 4). For patients with less than one urinary catheterization, the number of acupuncture treatment sessions was 2.8 ± 0.2 times and the cured rate was 96.4%. Only one (1.8%) patient showed no improvement. In contrast, for patients with two or more urinary catheterization, the number of acupuncture treatment sessions was 4.3 ± 0.3 times and the cured rate was 80.7% with 5 (8.8%) patients who had no improvement. Moreover, we further analyzed the number of acupuncture treatment sessions and efficacy rate in different diseases (Figure 5). The number of acupuncture treatment sessions was 2.6 ± 0.3 for postpartum, 3.3 ± 0.3 for gynecological surgery, 6.4 ± 1.4 for myelopathy, and 4.5 ± 0.9 for lumbar surgery, respectively. The cured rate was 100% for postpartum, 87.2% for gynecological surgery, 77.8% for myelopathy, and 50% for lumbar surgery, respectively. No side effects or secondary urinary tract infections occurred during the acupuncture treatment. Patients with improved and unchanged urinary retention were discharged with indwelling Foley catheters.

## 4. Discussion

Urinary retention usually occurs postpartum and after pelvic surgery (gynecological surgery and anorectal surgery), myelopathy, and lumbar spinal surgery [16]. The causes of urinary retention are multifactorial, and urinary retention can result from injury of the nerves between the bladder and the brain, which can cause the loss of bladder control. The dysfunction may involve the nerves that send messages back and forth, the nerves that control the muscles used for urination, or both. The dysfunctional nerves affect the normal use of the bladder, ureter, and urethra, causing bladder (detrusor) dysfunction or failure of pelvic floor relaxation leading to urinary retention. In addition, surgical anesthetics, local inflammation following surgery, age over 50 years, and relative immobility after surgery are also associated with higher rates of urinary retention [17, 18].

Acupuncture therapy is an important part of TCM. It has a curative effect and a long history in treating urinary retention. According to TCM theory, urinary retention belongs to the category of “LongBi.” Clinical characteristics of poor health such as (1) excessive blood loss or kidney qi injury during childbirth or operation, qi depletion with the blood, resulting in kidney qi deficiency, urinary adjustment failure, (2) depression after an operation causing liver qi stagnation, and (3) compression of the bladder for an extended period of time during labor causing bladder qi and blood stasis, leading to urinary retention. The mechanism of acupuncture is based on meridians. Stimulating the acupoints on the meridians can tonify kidney qi and stimulate micturition. In our study, we chose the CV6, CV4, CV3, and ST28 points as the primary local acupoints and the SP6, SP9, ST36, and GV20 points as the distal acupoints. According to acupuncture meridian theory, CV3 is the Mu point of the bladder meridian and CV4 is the intersection point of the conception vessel and the three yin channels of the foot, which is a key point for the clinical treatment of diseases of the urinary and reproductive system [19]. CV6 is associated with the conception vessel and functions to nourish vitality, benefiting the kidneys and strengthening essence and Yang. ST28 belongs to the stomach meridian of the foot-yangming and regulates water metabolism and promotes urination. The local acupoints above are all located near the bladder, and acupuncture at these points can clarify and regulate the bladder meridian and help to promote urination. SP6 is the intersection point of the liver, spleen, and kidney meridians, and SP9 is the he-sea point of the spleen meridian. Both acupoints are used to invigorate the spleen, regulate qi activity, and promote urination. GV20 is located at the midline vertex; it is the intersection acupoint of the governing meridian and the three Yang meridians and is known for lifting descending qi, promoting clear qi, relieving nervousness, and maintaining the water metabolism of the body. All of the above acupoints are commonly stimulated for the clinical treatment of urinary retention [20]. In addition, TCM meridian theory states that the ear is closely related to the viscera and the meridians. According to modern holographic theory, the auricle is a relatively independent holographic element, which is a microcosm of the whole human body. The auricle is associated with a corresponding acupoint area for each part of the human body, and stimulation of the auricle will be transmitted to the corresponding organs of the body through the holographic reflection path. Therefore, in this study, auricular acupoints (for the bladder and urethra) were also selected to improve clinical efficacy.

Consistent with other studies [21–23], our results showed that acupuncture reduced the residual urine volume in the bladder to improve urinary retention. Also, most of the patients were able to remove the catheter successfully with three or less acupuncture treatment sessions. We found the efficacy rate of acupunctured patients with postpartum urinary retention and gynecological surgery was better than with myelopathy and lumbar surgery. Especially for postpartum urinary retention, the cured rate was 100%. We speculated that the efficacy rate of acupuncture was related to the severity of injury. For the urinary retention caused by myelopathy, longer course of acupuncture treatment was needed. In this study, the cured rate of acupuncture patients with lumbar surgery was only 50%; we considered it may be due to the small sample.

Compared with conventional treatment in Western medicine, acupuncture is simple to perform, has a remarkable curative effect, and results in less adverse reactions. However, in this study, we found that 109 (96.5%) patients were not considered for acupuncture treatment until they required a reinsertion of catheter after the initial removal. Five patients underwent catheter removal and reinsertion more than 5 times, which seriously affected the quality of life of these patients, delayed additional treatment opportunities, and extended the length of hospitalization. Because of the long duration of urinary catheterization and repeated catheter removal and insertion, 27 (23.9%) hospitalized patients experienced urinary tract infections. There were no secondary urinary tract infections during the acupuncture treatmenit. It further indicates that acupuncture can be a good prophylaxis of recurrent urinary tract infections [24]. Moreover, we found that there was a significant difference in the efficacy rate between patients with one urinary catheterization and two or more. Together, it is strongly suggested that early application of acupuncture treatment should be considered in case of urinary retention, and repeated urinary catheter insertion and removal should be avoided.

This study has some limitations. First, the present study was retrospective and it did not have a control group. Second, data collection was based on the hospitalized patients who received acupuncture consultations. Most of the included patients were female who had received gynecology or obstetrics surgery. Thus, the data may not provide enough evidence supporting acupuncture in male patients with urinary retention. Therefore, the study population may not be representative of all urinary retention patients in the hospital. Third, as PUMCH is the national center for the diagnosis and treatment of complicated diseases, hospital beds are in high demand. If a urinary retention patient's primary disease was stable, they were discharged or transferred to a different hospital for rehabilitation with a catheter. Thus, some serious urinary retention patients, such as those with myelitis, may not have received acupuncture long enough, which could have influenced the efficacy analysis in this study. Therefore, multidepartment cooperation is suggested and a randomized controlled study with a larger sample size should be carried out in the future.

## 5. Conclusion

Our results suggest that acupuncture should be considered as an alternative treatment for urinary retention in clinical practice, and the repeated insertion and removal of urinary catheters should be avoided.

## Figures and Tables

**Table 1:** Patient characteristics.

Characteristics	Patients (*N* = 113)
Age (years)	43 (14–84)
Sex	
Male	6 (5.3%)
Female	107 (94.7%)
Duration of urinary catheterization (days)	12 (1–50)
Urinary catheter reinsertion	109 (96.5%)
Urinary catheter reinsertion times	
≥1 times	56 (49.6%)
≥2 times	57 (50.4%)
≥3 times	19 (16.8%)
≥5 times	5 (4.4%)
Catheter-associated urinary tract infection	27 (23.9%)

Data are presented as the number (percentage) or median (range) of patients.

**Table 2:** The causes of urinary retention.

The causes of urinary retention	Patients (*N* = 113)
Gynecological surgery	78 (69.0%)
Malignant tumor surgery	66
Benign tumor surgery	9
Pelvic organ prolapse surgery	3
Obstetric surgery	21 (18.6%)
Vaginal delivery	20
Cesarean delivery	1
Myelopathy	9 (8.0%)
Lumbar surgery	4 (3.5%)

Data are presented as the number (percentage) of patients.

## Data Availability

The data used to support the findings of this study are included within the article.
